# An investigation of sleep profiles in individuals with idiopathic scoliosis

**DOI:** 10.5935/1984-0063.20220038

**Published:** 2022

**Authors:** Yavuz Yakut, Zerrin Pelin, Gozde Yagci

**Affiliations:** 1 Hasan Kalyoncu University, Faculty of Health Sciences Physiotherapy and Rehabilitation Department - Gaziantep - Turkey.; 2 Hacettepe University, Faculty of Physical Therapy and Rehabilitation - Ankara - Turkey.

**Keywords:** Scoliosis, Sleep, Pain, Depression, Sleepiness

## Abstract

**Objective:**

Sleep behaviors have not been well investigated in individuals with idiopathic scoliosis (IS). This study aimed to investigate sleep quality and daytime sleepiness in individuals with IS and investigate the relationship between sleep parameters and curve magnitude, trunk deformity severity, pain, and emotional status in adolescents with IS.

**Material and Methods:**

Ninety-one participants between the ages of 10 and 19 years with IS were included. Sleep quality was evaluated using the Pittsburgh sleep quality index (PSQI), and daytime sleepiness was assessed with Epworth sleepiness scale (ESS). Pain was assessed using the short-form McGill pain questionnaire; the presence or severity of depressive feelings was evaluated using the Beck depression scale.

**Results:**

The majority of the participants (64.8%) had poor sleep quality, while daytime sleepiness was within normal limits in most participants (85.7%).The frequency of participants without pain (52.7%) was similar to participants with pain (47.3%). The prevalence of participants with depressed mood was 35.2%. Participants with poor sleep quality were more likely to have a higher sensorial index (*p*<0.001), higher total pain scores (*p*=0.001), and less lumbar axial rotation (*p*=0.046). Higher pain (r=0.391), depression scores (r=0.234), and lower lumbar axial trunk rotation (r=-0.317) were associated with increased daytime sleepiness.

**Conclusion:**

We observed poor sleep quality and an association with pain in patients with IS. Curve magnitude had no adverse effect on sleep quality or daytime sleepiness. Therefore, the sleep profile and its association with pain should be considered during the rehabilitation process in patients with IS.

## INTRODUCTION

Sleep is essential for achieving developmental milestones, including higher cognition, learning, and emotional regulation in adolescents^1^. The literature suggests that sleep deprivation can cause significant impairments such as sustained vigilance and attention, longer reaction times, slower cognitive processing speed, and lower academic achievement^2^. Given the critical role of sleep in high cognition, attention, memory, decision-making, emotional regulation, and vitality, it is essential to evaluate sleep parameters in adolescent care^3^.

In the adolescent period, higher cognition for academic demands (intense study pace and exams), extracurricular activities and socializing, adult relationships, and screen time on mobile phones or computers may interfere with sleep quality or optimal sleep duration^4^. A bidirectional relationship has been defined between insufficient sleep and depression, meaning that too much sleep or too little sleep is associated with depression^5^. In most musculoskeletal disorders such as osteoarthritis, fibromyalgia, chronic overuse syndromes, and musculoskeletal pain syndromes, poor sleep quality has been documented^6^. Given the physical and psychiatric symptoms caused by sleep disorders, these findings reveal how important it is to assess sleep-related problems in this age group.

Idiopathic scoliosis (IS) is a structural deformity of the spine, characterized by lateral deviation, axial rotation of the spine, and alterations in the physiological sagittal curves. IS is progressive and takes place in childhood, often between 8 and 18 years^7^. Biomechanical and morphological alterations are common in IS patients. These alterations include limb and trunk asymmetries, postural malalignment, compensatory movement patterns, morphological changes in ribs, pelvis, arm, and skull, and the loss of stability and mobility in the body^8,9^. In addition to physical problems, body image disturbance, body dissatisfaction, and self-consciousness that reduce the psychosocial quality of life are more common in patients with IS^10^.

Chronic back pain has been emphasized as a common symptom in patients with IS, with greater intensity and duration of pain than in healthy peers^7^. The etiology of back pain in IS has been reported as multifactorial and associated with psychological, biomechanical, and morphological parameters of scoliosis^11^. In the literature, chronic back pain has been found to lead to sleep disorders and reduced quality of life^12^. However, sleep can be underestimated in the scoliosis population because there are few studies in this area. Previously, sleep-disordered breathing increased abnormal respiratory patterns (apnea events and hypopnea), and insufficient ventilation during sleep was reported in patients with scoliosis^13^. In another study, poor sleep quality (76% prevalence) and a high prevalence of disability (52% prevalence) were linked to the negative impact of chronic pain in the IS population^14^. But studies were limited, and further studies are needed to assess outcomes related to sleep in individuals with IS.

In summary, like the above described, there is an association between physical disability, pain, psychological distress, and sleep disorders^12^. Such a relationship, however, has never been investigated for IS patients. We hypothesized that sleep function might be affected by curve magnitude, deformity severity, pain, or depressive symptoms in individuals with IS. This study aimed first to define sleep parameters such as sleep quality and daytime sleepiness in adolescents with IS. Second objective was to investigate the relationship between sleep parameters and curve magnitude, trunk deformity severity, pain, and emotional status in adolescents with IS.

## MATERIAL AND METHODS

### Participants and study design

This descriptive cohort study was conducted with 91 IS patients seen at the orthopedic department. The study was approved by the University’s Non-Interventional Clinical Research Ethics Committee. Overall, participants who met the criteria for this study signed an informed consent form, answered questionnaires, and underwent clinical assessments. This study’s inclusion criteria included a diagnosis of IS, age above 10 years, previous no history of treatment about scoliosis, the presence of lateral curve above 10 degrees of Cobb. Exclusion criteria included participants with a current or history of congenital, neurological, rheumatological, or cardiovascular disease, or psychotic disorders.

### Assessments

Participants’ demographics regarding age, sex, body weight, height, and body mass index were recorded. Assessment consisted of two parts. First part was scoliosis-specific clinical assessments performed by the orthopedic surgeon. Second part was face-to-face scale administration by the physical therapist. Patient clinical characteristics specific to scoliosis included curve pattern evaluation, curve intensity measurement with Cobb angle on a radiograph, and axial trunk rotation with a scoliometer. Sleep quality was evaluated using the PSQI and daytime sleepiness with the ESS. Pain was assessed with the short-form McGill pain questionnaire, and the presence or severity of depressive feelings was evaluated using the Beck depression scale (BDS). All assessments were performed at the first visit following the diagnosis, which took about forty-five minutes.

The curve pattern was determined as a single (thoracic or lumbar) or double curve. Cobb angle was measured on a frontal radiograph in standing as the angle of intersection of perpendicular lines from the endplates of the most tilted superior and inferior vertebrae^15^. Cobb angles were measured separately for the thoracic and the lumbar regions. The axial rotation angle of the trunk was measured in Adam’s forward bending test^16^. For that test, the concave notch of the scoliometer was placed over the spinous process of the apex vertebra and the angle of rotation was recorded. Axial trunk rotations were also measured separately for the thoracic and lumbar regions.

PSQI is an effective, reliable, and valid self-reported scale, which assesses sleep quality and disturbance during the past month^17^. PSQI consists of 19 items related to the following seven components: 1) subjective sleep quality, 2) sleep latency, 3) sleep duration, 4) habitual sleep efficiency, 5) sleep disturbance, 6) use of sleeping medication, and 7) daytime dysfunction. Each component was scored on a four-point ordinal scale from 0 to 3 (0 = no difficulty and 3 = severe difficulty). The total score ranged from 0 to 21. A total score above 5 was considered poor sleep quality, whereas scores equal to or below 5 indicated good sleep quality. The Turkish translation has shown acceptable reliability and validity to assess sleep problems^18^.

ESS is an 8-item self-reporting questionnaire to measure patients’ daytime sleepiness^19^. The scale consists of eight different questions involving everyday situations such as sitting or reading, watching television, driving, etc. Scores ranged from (0 ‘never’ to 3 ‘high chance’) to reflect the subjects’ probability. The total ESS scores ranged from 0 (less sleepy) to 24 (sleepier), with higher scores indicating more significant daytime sleepiness. Scores equal to or above 10 were considered excessive sleepiness, whereas scores below ten referred to hypersomnolence. The Turkish version of the ESS was a valid and reliable measure of daytime sleepiness in the Turkish population, suggesting use for clinical or research purposes^20^. Musculoskeletal pain was defined as diffuse pain or pain located in the back, joints, or limbs as previously described^21^. The presence of pain was recorded for all participants. Participants were asked to rate pain intensity of the back during the prior month. Pain intensity and characteristics for regional musculoskeletal pain were measured using the shortform McGill pain questionnaire^22^. The questionnaire consists of 15 descriptors with two summary scales, evaluating the sensory and emotional measures of pain and its subjective intensity. While the first 11 of the 15 descriptors were sensorial, the last four descriptors were emotional. Each of those four descriptors was ranked on an intensity scale of none (0), mild (1), average (2), and severe (3). The participants were asked to describe their pain by selecting one of the options. The total pain score was the sum of the ratings across all 15 descriptors and ranged from 0 (no pain) to 45 (intensive pain). The Turkish version of the short-form McGill pain questionnaire was determined valid and reliable in evaluating pain^23^.

The Beck depression scale assessed the presence and severity of depressive feelings^24^. It had 21 items, each with four possible answers (score: 0-3). Higher scores represented more severe symptoms and patient depression. In the general population, a Beck depression scale score of >11 was the cutoff for depression^25^. Scores of >11 were reported as indicative of depression in the general population.

### Statistical analysis

Data were analyzed using SPSS, version 20.0. Descriptive analysis using means and frequencies was undertaken for the characteristics of demographics, scoliosis-specific clinic, pain, sleep, and depression. Participants were grouped into two categories based on the cutoff PSQI point score of 5; participants with good sleep quality (cutoff score <5) and poor sleep quality (cutoff score >5). More specific comparisons regarding body mass index, gender, curve pattern, pain, sleep quality, daytime sleepiness, and depression were made between these two groups.

Comparisons between the two groups were performed using the independent sample t-test or chis quare test when appropriate. Bivariate correlation analysis was used to investigate the relationship between the Cobb angle, axial trunk rotation, pain, depression, and sleep variables (sleep quality-PSQI and daytime sleepiness-ES). The level of statistical significance was set at *p*<0.05.

## RESULTS

In total, 91 participants with IS were referred for a sleep assessment by an orthopedic doctor between October 2018 and January 2021. The participants included were aged 10 to 19 years and 86.8% female. The mean age was 13.63±2.03 years. The participants’ demographic and scoliosis-specific clinical characteristics are shown in Table 1. Participants had single thoracic, single lumbar or double curves. The majority (38.5%) were double curves. The mean Cobb angle of the participants was 28.9°±12.6 (range: 12° to 56°) for the thoracic region and 15.4°±8.9 (range: 12° to 45°) for the lumbar region. The mean axial trunk rotation angle of the participants was 8.6°±4.5 (range: 2° to 17°) for the thoracic region and 6.8°±3.3 (range: 2° to 19°) for the lumbar region. Forty-seven percent of participants had pain in the back region.

**Table 1:** Demographic and scoliosis-specific clinical characteristics of the participants.

	Participants (n=91)			
Characteristics	Mean	SD	Minimum	Maximum
**Age (years)**	13.63	2.03	10.0	19.0
**Gender n (%)** Girl		79 (86.8)		
Boy		12 (13.2)		
**Height (cm)**	161.81	11.04	122.0	185.0
**Body mass (kg)**	50.61	12.58	24.0	80.0
**Body mass index (kg/m^2^)**	19.24	3.32	10.39	27.55
**Curve pattern n (%)** Single thoracic		33 (36.3)		
Single lumbar		23 (25.3)		
Double curve		35 (38.5)		
**Cobb angle (°)** Thoracic	28.92	12.63	12.00	56.0
Lumbar	25.14	8.86	12.00	45.0
**Axial trunk rotation (°)** Thoracic	8.60	4.55	2.0	17.0
Lumbar	6.83	3.34	2.0	19.0

Note: Values are means ± standard deviations, or frequence (percentage); SD = Standard deviation.

Pain, sleep, and depression outcomes of the participants are reported in Table 2. The frequency of participants without pain (52.7%) was similar to those with pain (47.3%). The daytime sleepiness was within normal limits for most participants (85.7%). However, the majority of the participants had poor sleep quality (64.8%). Participants with depressed mood were 35.2%, whereas participants without depressed mood were 64.8%.

**Table 2:** Pain, sleep and depression outcomes of the participants.

Characteristics	Participants (n=91)			
	Mean	SD	Minimum	Maximum
**Pain n (%)** No pain		(52.7)		
Pain presence		(47.3)		
**McGill pain questionnaire** Emotional index	0.24	0.69	0	4
Sensorial index	2.04	3.13	0	16
Total pain rating index	2.29	3.43	0	17
**Epworth sleepiness scale** Total score	4.67	3.88	0	17
Cutoff n (%) <10 normal limits	(85.7)	n/a	n/a	n/a
>10 excessive sleepiness	13.14.3	n/a	n/a	n/a
**Pittsburg sleep quality index** 1) Subjective sleep quality	8.79	5.53	1.0	19
2) Sleep latency	1	0.56	0	2
3) Sleep duration	1.82	1.23	0	3
4) Habitual sleep efficiency	3.65	4.39	0	12
5) Sleep disturbance	0.20	0.46	0	2
6) Use of sleeping medication	1.33	0.60	0	2
7) Daytime dysfunction	0.01	0.11	0	1
Total score	0.94	0.89	0	3
Cutoff n (%) <5 good sleep quality	(35.2)	n/a	n/a	n/a
>poor sleep quality	(64.8)	n/a	n/a	n/a
**Beck depression scale** Total score	9.6	8.29	0	36
Cutoff n (%) <10 normal limits	(64.8)	n/a	n/a	n/a
>11 depression	(35.2)	n/a	n/a	n/a

Notes: Values are means ± standard deviations, or frequence (percentage); SD = Standard deviation.

A statistical analysis of the sleep quality categories based on PSQI showed that participants with poor sleep quality had higher pain prevalence than participants with good sleep quality (*p*<0.001). No difference was observed between the good sleep quality group and the poor sleep quality group concerning gender, curve pattern, daytime sleepiness for ESS, or depression for BDS variables (*p*>0.05) (Table 3).

**Table 3:** Frequency distribution and percentages of participants classified in groups based on Pittsburg sleep quality index cutoff scores.

Characteristics	Participants with good sleep quality (n=32)		Participants with poor sleep quality (n=59)		
	n %		n %		*p*-value
**Gender**					
Girl	31	96.9	48	81.4	0.50
Boy	1	3.1	11	18.6	
**Curve pattern**					
Thoracic	9	28.1	24	40.7	0.282
Lumbar	11	34.4	12	20.3	
Double curve	12	37.5	23	39	
**Pain**					
No pain	25	78.1	23	39	<0.001**
Pain presence	7	21.9	36	61	
**Epworth sleepiness scale**					
<10 normal limits	29	90.6	49	83.1	0.531
>10 excessive sleepiness	3	9.4	10	16.9	
**Beck depression scale**					
<10 normal limits	21	62.5	39	66.1	0.731
>11 depression	12	37.5	20	33.9	

Notes: Values are frequence (percentage); Significant results of group comparisons,

** *p*<0.001.

Patients with poor sleep quality had a significantly higher sensorial index (*p*<0.001) and total pain (*p*=0.001) scores in the short-form McGill pain questionnaire and less lumbar axial rotation than patients with good sleep quality (*p*=0.046). On the other hand, age, body mass index, Cobb angle, thoracic axial rotation, daytime sleepiness, and depression scores did not differ among the two sleep quality groups of participants (*p*>0.05) based on PSQI cutoff scores (Table 4).

**Table 4:** Comparison of curve magnitude, pain and depression scores among groups based on Pittsburg sleep quality index cutoff scores.

Characteristics	Participants with good sleep quality (n=32)		Participants with poor sleep quality (n=59)		
	Mean	SD	Mean	SD	*p*-value
**Age (years)**	13.76	1.46	13.58	2.23	0.725
**Body mass index (kg/m^2^)**	19.09	3.14	19.30	3.41	0.827
**Cobb angle (°)** Thoracic	>29.80	>9.43	27.67	13.01	>0.567
Lumbar	26.07	9.34	24.24	8.86	0.547
**Axial trunk rotation (°)** Thoracic	>9.0	>3.13	8.47	4.96	>0.741
Lumbar	8.25	4.04	6.04	2.64	0.046*
**McGill pain questionnaire** Emotional index	>0.22	>0.79	0.25	0.63	>0.816
Sensorial index	0.53	1.14	2.86	3.55	<0.001**
Total pain rating index	0.75	1.63	3.12	3.85	0.001*
**Epworth sleepiness scale**	3.88	3.55	5.10	4.01	0.151
**Beck depression scale**	9.59	8.77	9.61	8.09	0.933

Notes: Values are means ± standard deviations, or frequence (percentage) of the groups; Significant results of group comparisons,

* *p*<0.05,

** *p*<0.001

The bivariate correlations between Cobb angles, axial trunk rotations, pain and depression scores, and sleep-related outcomes are shown in Table 5. No significant association was found between the thoracic Cobb angle, axial trunk rotation, and PSQI and ESS scores. Increased lumbar Cobb angle (r=0.335, *p*=0.027) and lumbar axial rotations (r=-0.535, *p*<0.001) were associated with increased sleep quality; increased lumbar axial trunk rotation (r=-0.317, *p*=0.049) was also associated with reduced daytime sleepiness. Increased sensorial and total pain scores were associated with reduced sleep quality (r=0.333, *p*=0.001 and r=0.305, *p*=0.003) and increased daytime sleepiness (r=0.396, *p*<0.001 and r=0.391, *p*<0.001). Increased emotional pain (r=0.226, *p*=0.031) was associated with increased daytime sleepiness. Increased depression was associated with higher daytime sleepiness (r=0.234, *p*=0.026).

**Table 5:** Correlation between sleep scores and curve magnitude, pain and depression scores.

	Sleep scores			
Clinical outcomes	Pittsburg sleep quality index		Epworth sleepiness scale	
	r	p	r	p
**Cobb angles** Thoracic	-0.232	0.089	-0.293	0.030
Lumbar	**-0.335**	0.027*	-0.164	0.319
**Axial trunk rotation** Thoracic	>0.091	>0.551	-0.006	0.071
Lumbar	**-0.535**	<0.001**	**-0.317**	0.049*
**McGill pain questionnaire** Emotional index	>-0.035	>0.763	0.226	0.031*
Sensorial index	**0.333**	0.001*	**0.396**	<0.001**
Total pain rating index	**0.305**	0.003*	**0.391**	<0.001**
**Beck depression scale**	0.042	0.691	**0.234**	0.026*

Notes:

* Correlation is significant at the 0.05 level;

** Correlation is significant at the 0.01 level (2-tailed).

## DISCUSSION

This study provided descriptive information about sleep profiles in individuals with IS. The study showed that most participants with IS had poor sleep quality, regardless of the curve pattern. Participants with pain had an increased risk of sleep disorders, including poor sleep quality and excessive daytime sleepiness. Participants with depressed moods were more likely to be sleepy in the daytime. The most significant result of our study was that increased Cobb angle and axial rotation were associated with better sleep quality and less daytime sleepiness in participants with lumbar scoliosis. There was no relation between thoracic curve magnitude (Cobb angle and axial trunk rotation) and sleep quality or daytime sleepiness.

IS is the most common chronic condition, which causes musculoskeletal problems in adolescents^26^. Sleep is defined as physical and mental rest, during which the body recovers energy^27^. In chronic musculoskeletal conditions, sleep deprivation may occur, having a significant impact on a person’s quality of life. The psychophysical health of the individual depends on the quality and duration of sleep^27^. Additionally, a melatonin defect has been associated with IS as a hormonal developmental theory. Melatonin levels are known to diminish in sleep disorders, but there has been no evidence to suggest an association with IS^26^.

Poor sleep quality is defined by difficulties initiating or maintaining sleep or non-restorative sleep. Inadequate sleep adversely affects growth, behavior, and cognitive function in children^28^. In this study, we assessed sleep quality and excessive daytime sleepiness in participants with IS. The majority of the participants exhibited poor sleep quality (64.8%). Excessive daytime sleepiness has been associated with clinically significant distress or impairment in cognitive, social, occupational, or other vital areas^27^. Addressing those conditions is crucial when investigating the sleep profile.

Daytime sleepiness was within normal limits in 85.7% of the study participants. Previously, Li et al. (2018)^13^ described more respiratory events of apnea and hypopnea during sleep than healthy peers in individuals with IS. They observed that sleeping on the convex side of the thoracic curve results in a higher risk of sleep disorder breathing than sleeping on the concave side. In a recent study, MacKintosh et al. (2020)^28^ assessed sleep abnormality using polysomnography employed by Li et al. (2018)^13^. Their study found sleep abnormalities, including more frequent abnormal breathing during sleep and, less often, impairment in sleep quality in children with earlyonset scoliosis. They also reported abnormal sleep-related symptoms of snoring, arousals, hypoxemia, limb movements, and restless sleep. A case study of a patient with severe scoliosis treated with a brace showed sleep quantity and quality within normal trends^29^. The lack of relevant comparative data about the effect of IS on sleep made it difficult to compare the literature with our findings.

Previously, the prevalence of back pain in the adolescent IS population was reported to be 32%^30^. In a recent review 1-week prevalence was reported to be 17.7%, and 12-month prevalence was reported to be 33.6%^31^. In our study, 47% of participants experienced back pain in the past month, representing almost half of the participants with scoliosis. But the information about how long they experienced pain wasn’t asked to the participants. Therefore, it was not possible to assess if the pain was acute or chronic. Participants with poor sleep quality were more likely to experience pain. In the literature, a link between chronic pain and sleep deficiency in adolescents was 32%^32^. Chronic pain is associated with the disruption of physical activity, poor quality of life in youth, and emotional distress in both adolescents and parents. On self-reporting questionnaires, youth with painful conditions report more significant sleep disturbances than healthy populations, including difficulties falling asleep, maintaining sleep, feeling unrested, daytime sleepiness, sleepwalking, sleep-related anxiety, and sleep bruxism^33^. Pain should be considered a factor affecting the sleep profile when planning rehabilitation programs in individuals with IS.

Recent literature reported psychosocial difficulties in youth with IS such as negative body image, social limitation, low self-consciousness and self-esteem, altered mood, depressive symptoms, and anxiety^10^. Participants with depressed mood represented 35.2% of participants versus 64.8% without depressed mood. In contrast, Freidel et al. (2022)^34^ showed that 45% of 68 adolescents with IS had depressive symptoms. In another study, adolescents with IS treated operatively and conservatively also expressed depressive traits^35^. Furthermore, a depressed mood was more likely to increase daytime sleepiness, whereas not to have an effect on sleep quality in our study.

However, the current primary measures are not enough to explain this finding. Persistent sleep problems heighten the future risk for the development of psychiatric disorders, such as depression^36^. In a study on adolescents, sleep patterns were associated with positive and negative emotional status^37^. Adolescents with problematic sleep patterns may expose themselves to situations less conducive to healthy emotions (e.g., more negative and less positive situations). Meltzer et al. (2005)^38^ reported that depression and anxiety were related to daytime sleepiness but not total sleep time or sleep onset latency in adolescents with chronic musculoskeletal pain. Therefore, our study’s findings suggest future research on sleep patterns such as bedtime, sleep duration, fragmentation, waking-time, waking-time tiredness, and the role of these in the emotions of individuals with IS. Gender, curve pattern, and daytime sleepiness did not affect sleep quality.

Interestingly, greater curve magnitude (Cobb angle) and the severity of deformity (axial trunk rotation) were associated with better sleep quality and less daytime sleepiness in participants with lumbar scoliosis. Lumbar axial trunk rotation was more significant in participants with good sleep quality than participants with poor sleep quality. On the other hand, there was no correlation between thoracic curve magnitude and sleep parameters. MacKintosh et al. (2020)^28^ found that the magnitude of the preoperative coronal curve was not strongly associated with the severity of sleep-disordered breathing.

To our knowledge, this was the first study to examine the sleep profile of individuals with IS, as there is limited published literature examining sleep-related functions in that particular group. However, there were some limitations to our study. One potential limitation was the lack of a control group consisting of healthcare peers. Another limitation was the need for pediatric sleep assessment tools to define sleep profiles in pediatric populations. Sleep was evaluated with patient-reported outcome questionnaires. We did not aim to detect or diagnose any sleep disorders. However, our findings identified opportunities for future studies in which a laboratory-based sleep deprivation assessment could be performed on individuals with IS. More research is needed to investigate sleep-related functions in IS patients.

## CONCLUSION

In conclusion, poor sleep quality was common, whereas daytime sleepiness was primarily within the normal limits among individuals with IS. Participants with pain were more likely to experience poor quality sleep. A depressed mood seemed to increase daytime sleepiness. Based on this study, we suggest the use of sleep-specific patient-reported measures to assess sleep profiles in patients with IS. Poor sleep quality, pain and depressed mood may need to be considered as possible risk factors for future sleep problems, which may affect rehabilitation process in IS.
